# Statistical analysis plan for the Laser-1st versus Drops-1st for Glaucoma and Ocular Hypertension Trial (LiGHT): a multi-centre randomised controlled trial

**DOI:** 10.1186/s13063-015-1047-9

**Published:** 2015-11-11

**Authors:** Victoria Vickerstaff, Gareth Ambler, Catey Bunce, Wen Xing, Gus Gazzard, on behalf of the LiGHT Trial Study Group

**Affiliations:** Marie Curie Palliative Care Research Department, UCL Division of Psychiatry, University College London, 6th Floor, Maple House, 149 Tottenham Court Road, London, W1T 7NF UK; The Research Department of Primary Care and Population Health, University College London, Rowland Hill Street, London, NW3 2PF UK; Department of Statistical Science, University College London, Gower Street, London, WC1E 6BT UK; NIHR Biomedical Research Centre for Ophthalmology, Moorfields Eye Hospital and UCL Institute of Ophthalmology, City Road, London, UK

**Keywords:** Ophthalmology, open-angle glaucoma, ocular hypertension, randomised clinical trial, statistical analysis plan

## Abstract

**Background:**

The LiGHT trial (Laser-1st versus Drops-1st for Glaucoma and Ocular Hypertension Trial) is a multicentre randomised controlled trial of two treatment pathways for patients who are newly diagnosed with open-angle glaucoma (OAG) and ocular hypertension (OHT). The main hypothesis for the trial is that lowering intraocular pressure (IOP) with selective laser trabeculoplasty (SLT) as the primary treatment (‘Laser-1st’) leads to a better health-related quality of life than for those started on IOP-lowering drops as their primary treatment (‘Medicine-1st’) and that this is associated with reduced costs and improved tolerability of treatment. This paper describes the statistical analysis plan for the study.

**Methods/Design:**

The LiGHT trial is an unmasked, multi-centre randomised controlled trial. A total of 718 patients (359 per arm) are being randomised to two groups: medicine-first or laser-first treatment. Outcomes are recorded at baseline and at 6-month intervals up to 36 months. The primary outcome measure is health-related quality of life (HRQL) at 36 months measured using the EQ-5D-5L. The main secondary outcome is the Glaucoma Utility Index. We plan to analyse the patient outcome data according to the group to which the patient was originally assigned. Methods of statistical analysis are described, including the handling of missing data, the covariates used in the adjusted analyses and the planned sensitivity analyses.

**Trial registration:**

The trial was registered with the ISRCTN register on 23/07/2012, number ISRCTN32038223.

## Update

### Background

Glaucoma is a common, irreversible, optic neuropathy that affects the vision of predominantly older adults and slowly progresses over a period of years. In the United Kingdom glaucoma affects more than half a million individuals, and more than a quarter a million are over the age of 65 [1]. Glaucoma significantly reduces the quality of life, which is worse with more severe field loss [2–5].

Ocular hypertension (OHT) is a state of raised intraocular pressure (IOP) without optic nerve damage, which progresses to open-angle glaucoma (OAG) in some patients [6, 7]. Approximately 1.2 million individuals have raised IOP in the UK [8].

Progressive visual loss can be halted or slowed at all stages of glaucoma. IOP is the only modifiable risk factor proven to alter the disease course and thus the associated morbidity. Laser, medicines and surgery can all successfully reduce IOP [9–13]. If medical treatment is selected, the installation of drops needs to be lifelong. Surgery, while effective, carries significant operative risks and is usually reserved for those who continue to lose vision despite other treatments. It also has a significant failure rate, often causes permanent ocular discomfort and rarely chronic pain [14, 15].

Initial treatment with laser trabeculoplasty (LT) potentially offers a ‘drop-free window’ of several years, removes concerns about compliance and probably reduces the need for multiple drops even years later. Even when insufficient as a sole therapy, LT reduces the intensity of subsequent medical treatment and, possibly, the need for later surgery. More effective long-term IOP control in glaucoma leads to better visual outcomes and less blindness. Drop usage is itself associated with poorer HRQL in glaucoma patients through their side effects, the burden of instilling them several times a day and the need for hospital visits [16]. A single outpatient treatment is likely to be more acceptable to patients than daily self-administration of eye-drops for many years.

The ‘LiGHT’ (Laser-1st versus Drops-1st for Glaucoma and Ocular Hypertension) Trial is a multi-centre randomised controlled trial comparing health-related quality of life in patients with newly diagnosed open-angle glaucoma or ocular hypertension. The objective of this study is to establish whether initial treatment with selective laser trabeculoplasty (SLT) of patients with newly diagnosed open-angle glaucoma or ocular hypertension is superior to the current standard initial treatment with topical medication alone in terms of better health-rlated quality of life (HRQL) at 3 years; lower cost; equally good intra-ocular pressure control with less need for topical medication and better patient tolerance.

To prevent outcome reporting bias and data-driven analysis results, the International Conference on Harmonisation (ICH) guidelines state that the primary analysis should be pre-specified [17]. Here, we describe the statistical analysis plan that will be used to produce the main trial results. This plan has been finalised while the data collection in the LiGHT trial is still on-going. Full details regarding the rationale and design of the study are given in the study protocol.

### Study design

The LiGHT trial is an unmasked, multi-centre, randomised controlled trial of initial selective laser trabeculoplasty versus conventional medical therapy.

Patients have been recruited from six centres in the UK. After obtaining written informed consent from each participant, patients are being randomly assigned to either medicine-first or laser-first treatment. Participants are randomised in equal proportion using a web-based randomisation service provided by a specialist company to achieve full allocation concealment. Block randomisation, with random block sizes, is used to randomise at the level of the patient and is stratified by diagnosis (OHT/OAG) and treatment centre. The trial duration per participant is 3 years. Participants will complete questionnaires at baseline and at 6-month intervals up to and including 3 years.

#### Main hypothesis

For patients with ocular hypertension (OHT) or open-angle glaucoma (OAG), use of SLT as the primary treatment (‘Laser-1st’) to lower IOP is associated with better health-related quality of life compared to the use of IOP-lowering drops as the primary treatment (‘Medicine-1st’).

#### Secondary hypotheses

Patients started on SLT as their primary treatment (‘Laser-1st’) have reduced costs and improved tolerability of treatment compared to patients started on IOP-lowering drops as their primary treatment (‘Medicine-1st’).

#### Sample size

The sample size for the study is 718 participants. This number of participants is required to detect a difference of 0.05 on the EQ-5D-5L between the two arms at 36 months, assuming a common standard deviation of 0.19 [18], 90 % power and using a two sample t-test at the 5 % significance level. This sample size assumes a 15 % loss to follow-up.

#### Ethics

Ethical approval has been obtained from the City Road and Hampstead REC (12/LO/0940).

### General considerations

#### Levels of confidence and P values

Statistical tests and confidence intervals will be two-sided. Estimates of treatment effects will be presented with 95 % confidence intervals and significance will be considered at the 5 % level.

#### Protocol violations and exclusions from the study

All patients will be analysed in the treatment arm to which they were randomised, and all patients will be included, whether or not they received the allocated treatment. Protocol deviations and exclusions, including reasons for any such exclusions, will be reported for each arm of the trial.

#### Unit of analysis

The unit of analysis is the patient. If the patient has both eyes in the study, we will use the worst eye at baseline for severity and baseline IOP covariates. The worst eye is defined using the mean deviation (MD) at baseline, with the worse eye having the most negative MD. In the case where the MD is the same in both eyes, to two decimal places, we will define the worst eye as the one with the highest IOP. If the patient only has one eye included in the study, we will use the severity and baseline IOP of this eye.

#### Unadjusted and adjusted analyses

For each outcome variable, the covariate-adjusted analysis will be designated the primary analysis. The covariate-adjusted analysis will incorporate the following covariates: the randomisation factors (severity and centre), baseline IOP, the baseline value of the corresponding outcome and whether the patient has one or two eyes affected at baseline.

#### Missing data

In the event of non-response after each follow-up, the subjects will receive two written reminders and then one telephone follow-up.

Even with the reminders, some loss to follow-up is expected over 36 months. The proportion of participants missing each outcome will be summarised in each arm and at each time point.

The main analysis of the primary outcome uses the data at 36 months. If this is missing, we will impute this missing data using the outcome measured at month 30. Further analyses based on imputed data will be reported as part of the sensitivity analyses (described later). The presentation of all findings will be in accordance with the latest CONSORT statement.

#### Interim analyses and stopping rules

Interim analyses may be conducted for the data safety and monitoring committee (DMEC) if requested as per the agreed terms of reference, but there are no planned interim analyses to examine efficacy and hence no adjustment to inflate the sample size.

We have not defined stopping/discontinuation rules for early termination of the trial because the two treatment pathways are designed to generate equivalent attainment of treatment targets, with possible differences in treatment-related HRQL and cost but not vision. No difference in safety outcomes is expected, and should the data monitoring committee request interim analyses, these will be supplied.

#### Start of data analysis

The analysis of the main trial data for publication purposes will begin once the final randomised patient has reached the 36 months follow-up and the data has been cleaned.

### Proposed analyses

#### Baseline

The baseline variables will be as follows: age, sex, ethnicity, centre, highest educational achievement, employment, diagnosis (open-angle glaucoma, ocular hypertension), intraocular pressure, habitual visual acuity (the vision obtained using the current spectacle or contact lens correction), distance visual acuity, refraction, medication use (statins, systemic beta blockers, systemic calcium channel blockers, and ace inhibitors), and general clinical factors (asthma, hypertension, diabetes, angina, and cardiac arrhythmia).

For eye-based characteristics, all eligible eyes will be described using two approaches: 1) left/right eye and 2) worst/best eye.

The baseline characteristics of each group will be summarised as the mean and standard deviation for continuous, symmetric variables, medians and inter-quartile ranges for continuous, skewed variables and frequencies and percentages for categorical variables. The baseline summaries will also be presented by centre. These summaries will be based on observed observations only and the number of missing observations will be reported. No significance testing will be used.

#### Trial profile

The flow of study participants will be displayed in a CONSORT diagram. The diagram will include the number of eligible patients, number of patients agreeing to enter the trial, then by treatment arm: the number of patients who are compliant/non-compliant, the number continuing through the trial, the number withdrawing at each time point, the number lost to follow-up at each time-point and the numbers excluded/analysed.

Compliance will be assessed by whether a patient received the randomised treatment and how compliant they were with respect to the prescribed treatment (for example, taking drops).

#### Primary outcome

The primary outcome measure is health-related quality of life (HRQL) measured using the EQ-5D-5L at 36 months. The EQ-5D-5L is a standardised measure of health status, applicable to a wide range of health conditions and treatments. Its name means ‘EuroQol- 5 Dimensions - 5 Levels’. It comprises five dimensions of health: mobility, ability to provide self-care, ability to undertake usual activities, pain and discomfort, and anxiety and depressions. The study is powered to detect a difference between trials arms in the EQ-5D-5L of 0.05 at 36 months.

The EQ-5D-5L will be analysed using regression methods (analysis of covariance). This will be an adjusted analysis using the covariates specified earlier (see General considerations section).

#### Secondary outcomes

The secondary outcome measures are as follows: 1) Glaucoma Utility Index (GUI), 2) Glaucoma Symptom Scale (GSS), 3) Glaucoma Quality of Life-15 (GQL-15), 4) objective measures of pathway effectiveness and visual function 5) side effects and adverse events, and 6) concordance/compliance.

Details on the secondary outcomes can be seen in Table 1. Further details can be seen in the protocol (Version 3.0, 20 May 2015).

**Table 1:** Secondary outcomes

Outcome	Description
Glaucoma Utility Index (GUI)	A glaucoma-specific treatment-related quality of life specifically designed to capture the impact of glaucoma treatment and disease severity on HRQL. This is the main secondary outcome.
Glaucoma Symptom Scale (GSS)	A patient-reported disease and treatment related symptoms questionnaire.
Glaucoma Quality of Life-15 (GQL-15)	A patient-reported visual functioning questionnaire.
Client Services Receipt Inventory (CSRI)	A validated method of collecting healthcare cost data. This data will be analysed by the health economists.
Objective measure of pathway effectiveness	Efficacy and intensity of the treatment pathways will be assessed at 3 years.
Concordance/Compliance	A pair of questions will be asked about drop usage and compliance: 1) ‘Over the past month, what percentage of your drops do you think you took correctly?’ 2) On a Likert scale, participants will be asked to respond to the following statement): ‘I’m the sort of person who follows doctors’ orders exactly’.
Adverse events	Adverse events possibly associated with treatment will be recorded. Participants will be asked about possible treatment-related side effects using a simple standardised series of closed and open questions at each visit.

The secondary outcomes will be analysed using the appropriate regression methods for the type of outcome. Results from all secondary analyses will be presented as estimates with confidence intervals and treated as exploratory.

#### Data at all time-points

We will use mixed effect models [19], using all patient outcome data over the 36-months, to investigate how the primary and secondary outcomes change over time. Such models allow analysis of repeated outcome measurements data (recorded every 6 months) while taking into account the correlation between measurements from the same patient. By using interaction terms between randomisation group and time, we will to investigate differences between groups over time. Regression splines will be used to explore non-linear trajectories, if such exist.

We will also use a similar mixed effect model using all patient data over the 36 months to evaluate the treatment effects at 36 months by using the exact times the questionnaires were completed.

Lastly, using all the patient data over the 36 months, we will use a mixed effects model to explore the average treatment effect over the 36 months.

#### Analysis of missing data

Potential bias due to missing data will be investigated by comparing descriptively the baseline characteristics of the trial participants with complete follow-up measurements to those who have incomplete follow-up or no outcome data. Reasons for withdrawal from treatment will be summarised. Patients who are compliant with their treatment plan will be compared descriptively with non-compliant patients in terms of their baseline characteristics.

#### Analyses of homogeneity

In order to explore the homogeneity (or otherwise) of the intervention effect on the primary outcome, we will examine the treatment effect across the following: age (as a continuous measure); severity of glaucoma (using the two groups OHT/OAG used during randomisation process); baseline IOP (as a continuous measure) and sex. The results from these analyses will be treated as exploratory. The estimates will be reported descriptively with 95 % confidence intervals. P-values will not be reported.

#### Sensitivity analyses

The following sensitivity analyses will be performed.

First, we will run sensitivity analyses that adjust for variables associated with missingness. We will perform logistic regression analyses (with missing yes or no as outcome) to identify predictors of missing data. If predictors associated with both missing data and outcomes are found, we will re-fit the primary analysis model, adjusting for these predictors of missingness.

Second, we will use a multiple imputation approach. The imputation model will include the outcome of interest, socio-demographic variables and any other variables potentially related to missingness and health related quality of life (HRQL). The imputations will be performed separately by treatment arm.

The number of clinic visits, intensity of treatment and treatment compliance will be explored. If necessary, sensitivity analyses adjusting for these factors will be carried out. For example, in addition to the primary analysis, we may perform analysis using the ‘per-protocol’ approach.

#### Health economic analyses

As stated in the trial protocol, there will also be health economic analyses. The details of these analyses are documented separately.

#### Current trial status

Recruitment has now finished. The final participant will be followed up in October 2017.

## Abbreviations

DMEC: data safety and monitoring committee
EQ-5D-5L: EuroQol-5 Dimensions-5 Levels
GQL-15: Glaucoma Quality of Life-15
GSS: Glaucoma Symptom Scale
GUI: Glaucoma Utility Index
HRQL: health-related quality of life
ICH: International Conference on Harmonisation
IOP: intraocular pressure
LT: laser trabeculoplasty
MD: mean deviation
OAG: open-angle glaucoma
OHT: ocular hypertension
SLT: selective laser trabeculoplasty
