# Erratum to Statistical analysis plan for the Laser-1st versus Drops-1st for Glaucoma and Ocular Hypertension Trial (LiGHT): a multi-centre randomised controlled trial

**DOI:** 10.1186/s13063-017-2033-1

**Published:** 2017-07-11

**Authors:** Victoria Vickerstaff, Gareth Ambler, Catey Bunce, Wen Xing, Gus Gazzard, on behalf of the LiGHT Trial Study Group

**Affiliations:** 1 Marie Curie Palliative Care Research Department, UCL Division of Psychiatry, University College London, London, UK; 2 The Research Department of Primary Care and Population Health, University College London, London, UK; 3 Department of Statistical Science, University College London, London, UK; 4 NIHR Biomedical Research Centre for Ophthalmology, Moorfields Eye Hospital and UCL Institute of Ophthalmology, London, UK

## Erratum

In the publication [1] there were errors in the acknowledgments. An investigator’s name was not spelt correctly and the Department of Health disclaimer was not included.

The investigator name was spelt as:

Haogang Zhou

The correct version is:

Haogang Zhu

The correct acknowledgements are:

We would like to acknowledge the following: the LiGHT Trial Study Group (Gareth Ambler, Keith Barton, Rupert Bourne, David Broadway, Catey Bunce, Marta Buszewicz, Amanda Davis, Anurag Garg, David Garway-Heath, Gus Gazzard, Rachael Hunter, Yuzhen Jiang, Evgenia Konstantakopoulou, Sheng Lim, Joanna Liput, Timothy Manners, Stephen Morris, Neil Nathwani, Gary Rubin, Nicholas Strouthidis, Victoria Vickerstaff, Sarah Wilson, Richard Wormald, Haogang Zhu); Moorfields Eye Hospital NHS Foundation Trust for acting as sponsors for the study; Moorfields Biomedical Research Centre for providing R&D support; and the PRIMENT Clinical Trials Unit for providing support in the design and conduct of the trial.

This project was funded by the NIHR [HTA] (09/104/40-LiGHT). The views expressed are those of the authors and not necessarily those of the NHS, the NIHR or the Department of Health.
